# Online compassion-based self-help for depression in people with skin conditions: a feasibility study

**DOI:** 10.1186/s40814-024-01486-4

**Published:** 2024-04-16

**Authors:** Elaine N. Clarke, Paul Norman, Andrew R. Thompson

**Affiliations:** 1 Department of Psychology, Sociology & Politics, Sheffield Hallam University, Heart of the Campus, Collegiate Crescent, Sheffield, S10 2BP UK; 2 Department of Psychology, University of Sheffield, Sheffield, UK; 3 School of Psychology, Cardiff University, Cardiff, UK; 4 Cardiff and Vale University Health Board, Cardiff, UK

**Keywords:** Self-compassion, Depression, Self-help, Online, Feasibility, Acceptability, Skin

## Abstract

**Background:**

There is a need to develop psychological interventions for depression in people with skin conditions. This study aimed to investigate the acceptability, feasibility, and effects of an online compassion-based self-help intervention for depression in people with skin conditions.

**Methods:**

Adult participants (*n* = 34) with skin conditions and mild-moderate depressive symptoms were invited to undertake a six-week, compassion-based online intervention for depression with email support. Engagement with the intervention was monitored, along with time spent facilitating the intervention, and participant feedback was collected each week and post-intervention. Pre-post changes in depression, self-compassion and dermatological quality of life were also assessed.

**Results:**

The intervention was started by 25 participants and completed by 13. Feedback scores indicated that the website was evaluated positively and that the sessions had positive impacts on participants. Participants appreciated the skin-specific aspects of the intervention but varied as to which of the compassion-based exercises they found helpful. The online intervention was feasible to provide and facilitate, and treatment completers showed improvements in depression, quality of life and self-compassion.

**Conclusions:**

The online compassion-based intervention holds promise as a treatment for depression in people with skin conditions. Recommendations are made for future research and further development of the intervention.

**Trial registration:**

This study was registered at ClinicalTrials.gov on 21 October 2019, NCT04132973.

**Supplementary Information:**

The online version contains supplementary material available at 10.1186/s40814-024-01486-4.

## Key messages regarding feasibility

- What uncertainties existed regarding the feasibility?
  It was previously uncertain whether an online, skin-specific, compassion-based, guided self-help intervention for depression would be perceived as acceptable to people with skin conditions and whether it would be feasible to provide the intervention in this way.
- What are the key feasibility findings?
  The content and format of the intervention were perceived as acceptable by users, although suggestions for improvement were noted. It was feasible to provide the intervention online and required minimal administration time.
- What are the implications of the feasibility findings for the design of the main study?
  Further action to engage participants at the beginning of the intervention is needed, and efforts should be made to investigate the reasons for discontinuation of the programme. The intervention should be modified to allow users flexibility about how they use it. There is a need to test the intervention in an adequately powered RCT.

## Background

### Psychological treatments for people with skin conditions

Skin conditions can have a considerable impact on people’s lives, with common areas of difficulty including work, leisure, socialising and relationships [1]. Skin conditions can also have a detrimental impact on self-image and mental health [2]. Studies of dermatology patients have found significantly higher rates of depression, anxiety and suicidal ideation than people without skin conditions [3]. However, few psychological treatments are available specifically for people with skin conditions: the most commonly used treatments are habit reversal, cognitive behavioural therapy (CBT), arousal reduction and combined techniques [4]. Habit reversal focuses on reducing itch/scratch cycles, to improve the condition of the skin in pruritic conditions. CBT focuses on changing unhelpful thoughts and behaviours associated with the skin condition, while arousal reduction focuses on reducing physiological arousal through techniques such as relaxation or meditation. Although these treatments have medium-sized effects on psychosocial outcomes (*g* = 0.53), there is a need to develop further interventions to extend the options available for people with skin conditions [4].

Psychological interventions that have been specifically developed for people with skin conditions often focus on anxiety rather than depression (e.g. [5, 6]), as it has been noted that people with conditions that affect their appearance can experience similar difficulties to people with social anxiety disorder, such as fear/avoidance cycles about public scrutiny [7]. However, not all people with skin conditions experience difficulties with (social) anxiety as their primary psychological problem. Clinical depression has been found to be present in 10.1% of dermatology patients, with an odds ratio of experiencing depression of 2.4 compared to controls [3]. This indicates a need for psychological interventions for people with skin conditions that focus specifically on depression.

## Treatment of depression and physical health conditions

Guidelines for the treatment of depression recommend the use of a stepped-care approach [8, 9], where the least restrictive treatment that is likely to improve health is first recommended, and then more intensive treatment is provided as necessary [10]. One type of recommended low-intensity intervention is guided self-help, in which patients receive self-help materials and limited support from a trained practitioner. As well as being cost-effective, guided self-help can enable patients to access an intervention despite geographic or time restrictions, particularly if the guidance is provided remotely.

However, interventions for depression need to acknowledge the presence and impact of chronic physical health conditions when co-morbidity occurs, as it is recognised that depression and chronic physical health problems can adversely affect each other: the impacts of chronic physical health problems increase the risk of depression, while depression can exacerbate pain and distress in people with physical illnesses [9]. Self-help interventions for depression typically use ready-made resources (e.g. written case examples, psychoeducation materials, worksheets), but it is important that such resources are tailored for people with specific health conditions to help them engage with the intervention [11]. For example, including normalising information about the common impacts of the individual’s physical health condition is likely to be de-shaming and increase the perceived credibility of the intervention. Thus, there is a need to develop specific self-help resources for depression in people with skin conditions. Skin-specific self-help resources are likely to be particularly valuable as many people with skin conditions manage alone or with the help of their GP only, and even for people who are referred to dermatology services, access to psychological support is limited [1].

## Compassion-based interventions

In recent years, there has been increasing interest in the use of compassion as a therapeutic tool. Definitions of compassion vary but generally include the concepts of noticing suffering and trying to alleviate it [12]. Corresponding to the different theoretical approaches to compassion, various compassion-based interventions have been developed, which have been shown to have medium-sized effects on depression (*d* = 0.64, *k* = 9, *n* = 470) [12]. While this meta-analytic finding is predominantly based on the results of group interventions, compassion-based self-help has also been shown to improve depression in students [13], self-critical people [14, 15], people with low–moderate wellbeing [16], and perinatal and intending-to-become-pregnant women [17].

Being self-compassionate may be a particularly adaptive strategy for people with skin conditions, as for some people, skin symptoms and/or negative social reactions can trigger negative self-evaluations [2, 18]. Furthermore, there is evidence that self-compassion may protect against depression in people with skin conditions [19] and facilitate adjustment to chronic skin conditions [20]. Previous research on compassion-based self-help in people with skin conditions has found it to reduce shame [21], negative affect [22] and depression [23]. In the latter study, soothing breathing self-help materials were compared to a waitlist control condition among a sample of people with heterogeneous skin conditions; the soothing breathing self-help led to reduced depression two weeks later [23]. However, the findings also suggested a need for further intervention development work, as only 35% of participants agreed that they would recommend the self-help materials to a friend or family member, which indicates scope to improve the materials. The self-help booklet was adapted from materials developed by Muftin et al. [21] and, while designed for people with skin conditions, focused on appearance-related distress and did not mention other common impacts of skin conditions, such as pain, itch, treatment burden, and restriction of valued activities. Thus, incorporating more content relating to common skin-related difficulties may be beneficial, as this may help users relate to and engage with the material [11]. In addition, Hudson et al. [23] recommended investigation of alternate methods of treatment delivery, as some participants had trouble accessing the self-help materials via email. This was eventually resolved by putting the materials online, which suggests that making the materials available online from the outset would be useful for participants. Therefore, a logical next step in the development of this intervention is to investigate the acceptability of an updated version of the self-help materials and the feasibility of providing them online.

Two other previous studies that have investigated the effects of compassion-based self-help on depression in people with skin conditions should be noted. Both had methodological limitations that informed the current study. In the first, Kelly et al. investigated self-soothing techniques based on Compassionate Mind Training [24] with people with acne [25]. Although the self-soothing self-help reduced shame at two weeks compared to the control condition, depression was not reduced. However, the self-soothing intervention comprised compassionate imagery, letter writing and self-statements, and it is possible that a multi-component compassion intervention may not be able to reduce depression in so short a time, given the lack of time for participants to practise any of the techniques sufficiently. This suggests that research investigating the effects of multi-component interventions on depression should do so over a longer period of time than two weeks.

In the second, D’Alton et al. found that a self-help version of mindfulness-based self-compassion therapy had no significant effect on symptoms of depression over eight weeks in people with psoriasis [26]. Despite this, participants reported finding the compassion-based self-help satisfactory and beneficial, and that they would recommend it to others. This provides some evidence of the acceptability of compassion-based self-help among people living with skin conditions and suggests that this population may value such a resource. The lack of a significant effect on depression in this study was likely due to a floor effect, as people with suspected depressive disorders were excluded. None of the interventions in the study were effective for depression, contrary to expectations, which suggests that research on interventions that aim to reduce symptoms of depression should ensure that participants experiencing such symptoms are included.

## The current study

The Medical Research Council recommends the use of feasibility studies as part of a systematic approach to developing, evaluating and implementing complex interventions [27]. Feasibility studies are preliminary pieces of research that aim to explore certain aspects of the intervention that will inform future clinical trials [28]. The current study sought to build on previously researched compassion-based self-help interventions for people with skin conditions [23, 25, 26], by addressing their limitations of including only participants who were not experiencing symptoms of depression, using self-help over a short (two week) intervention period, and using self-help materials that were difficult to access and not perceived as acceptable to many participants. Therefore, the current study investigated a compassion-based guided self-help intervention, delivered online over six weeks, for people with skin conditions who were experiencing depressive symptoms. There were three main aims. First, the study aimed to explore whether the intervention was perceived as acceptable to people with heterogeneous skin conditions, in terms of retention rates and explicit feedback. Second, the study aimed to investigate the feasibility of providing online compassion-based self-help and email guidance. Finally, the study aimed to assess changes in depression, self-compassion and skin-related quality of life to give an estimate of likely effect sizes for future research.

## Method

### Design

Given that the current study was an acceptability and feasibility study of a novel intervention, an uncontrolled pre–post design was used.

### Participants

Participants were adults who reported living with a diagnosed skin condition and experiencing symptoms of depression, as identified by scoring 10–20 on the depression subscale of the Depression Anxiety Stress Scales (DASS) [29], which represents mild–moderate levels of depressive symptomatology. In addition, participants needed to be aged 18 or over and have sufficient English language ability to read self-help materials and complete self-report questionnaires. People who reported having concurrent psychological treatment for a mental health condition, a diagnosed serious mental illness (e.g. bipolar disorder or psychosis) or a diagnosed drug/alcohol addiction were excluded, as it was expected that these factors might reduce participants’ abilities to engage with and benefit from the intervention.

### Procedure

Participants were recruited online, from November 2019 to January 2020, using adverts on the social media accounts and/or websites of organisations offering support for people with skin conditions: the British Association of Dermatologists, the British Skin Foundation, Lupus UK, the National Eczema Society, Pem Friends (pemphigus and pemphigoid), the Psoriasis Association, and TalkHealth’s acne forum. Social media adverts were also displayed on the lead researcher’s social media accounts and email adverts were sent to student members of the University of Sheffield’s volunteers list. Data was collected using Qualtrics, an online survey provider. People who were interested in taking part accessed the online participant information sheet, then completed the consent form, answered screening questions and completed the baseline measures, which consisted of demographic and clinical questions, and measures of depression, self-compassion and dermatology quality of life. Individuals who did not consent or who did not fulfil the inclusion criteria based on their answers to the survey questions were automatically redirected to an ‘end of study’ webpage. Signposting information about mental health resources and emotional support for skin conditions was provided to all participants. Eligible participants were invited to provide their contact details at the end of the baseline survey. Participants who did so were then registered on the study website by the lead researcher and sent login details by email, with instructions to begin the intervention by accessing the first session. Requiring participants to log in to the study website meant that engagement with the intervention could be monitored without involving any third parties for website traffic statistics and meant that each participant’s use of the website could be tracked over multiple devices. The homepage and contact page of the study website could be accessed without login details so that any participants who lost their login details could contact the researcher for assistance.

At the end of the first session, participants were asked to provide feedback on the session via an online survey. Participants received an automated email three days after completing the baseline measures to remind them to practise the homework exercise, or to go through the session if they had not already done so. One week after completing the baseline measures, participants received an automated email with a link to the second session of the intervention. Automated emails continued to be sent in this way, with homework reminders and links to the subsequent sessions of the six-session programme. The emails also encouraged participants to contact the lead researcher if they required further support with the self-help materials. The lead researcher responded to any such emails individually. One week after the last session, participants were invited to provide feedback via Qualtrics about the guided self-help intervention overall, and to complete the depression, self-compassion and dermatology quality of life outcome measures again. One week later, a reminder email was sent to participants who had not yet completed the overall feedback and outcome measures.

### Intervention

The guided self-help intervention, named ‘Compassion for Skin Conditions’, consisted of six online sessions of self-help information for participants to work through, plus activities to carry out in-between sessions. The intervention was developed using a self-help leaflet used in previous studies of compassion exercises for people with skin conditions [21, 23] and explanations of the Compassion Focused Therapy (CFT) approach as described by Gilbert [30]. The intervention contained psycho-education material, self-monitoring, and compassion-inducing exercises from CFT [31], including audio recordings adapted from those used in a previous study [13]. Session 1 explained the brain’s three affect regulation systems (threat/protection, drive/excitement, and soothing/contentment) and introduced soothing rhythm breathing as a way of activating the soothing/contentment system. Session 2 provided further explanation of how internally generated thoughts (especially self-criticism) can keep the threat/protection system activated. Session 3 introduced the use of compassionate imagery to generate internal signals of compassion. Session 4 presented an exercise for writing a self-compassionate letter, based on one used in previous research [32]. Session 5 presented the qualities of compassion (wisdom, strength and commitment) and the need for compassionate actions. It also included exercises for developing one’s compassionate self and addressing self-criticism. Session 6 focused on planning for the future (relapse prevention). Further details of the intervention are shown in Table 1. The session content and homework exercises built on previous sessions and homework, to give a clear sense of progression through the intervention. Examples relating to skin conditions were given throughout the self-help materials, including quotes from previous research participants [20], under pseudonyms.

**Table 1:** Components of the Compassion for Skin Conditions self-help intervention

Session number	Session content	Homework activity
1	Normalising the difficulties of living with skin conditions. Explanation of the three systems of emotions (threat/protection, drive/excitement, and soothing systems) and the soothing system as the regulator of threat/protection system. Audio recording (and text version) of soothing rhythm breathing practice.	Practise soothing rhythm breathing, e.g. 5 min daily.
2	Tricky brain explanation (emotions and cognitive abilities). Vicious cycle of thoughts and emotions. Example thought record.	Notice self-criticism: fill in your own examples on the thought record (situations, emotions and thoughts). Continue breathing practice.
3	Example of how the emotional tone of self-talk can affect our feelings. Audio recording (and text version) of ‘compassionate other’ imagery.	Practise ‘compassionate other’ imagery, e.g. 5 min daily.
4	Compassionate writing instructions. Example compassionate letter to oneself.	Write a compassionate letter (5–15 min).
5	Explanation of qualities of compassion (wisdom, strength/authority, and commitment). Audio recordings (and text versions) of ‘building the compassionate self’ imagery and ‘addressing self-criticism’.	Practise ‘compassionate self’ imagery, e.g 5 min daily.
6	Explanation of why a written relapse prevention plan (summary of learning points) is helpful. Example relapse prevention worksheet.	Complete the relapse prevention worksheet.

Prior to recruiting participants, expert feedback on a draft version of the website was sourced through personal contacts and social media. Feedback was gathered from four people with skin conditions, and changes to the website deemed necessary based on their feedback were made—these changes were mostly typographical and elucidated certain points more clearly. In addition, guidance on the content of the intervention was provided by an expert in CFT.

### Measures

#### Demographics

As part of the baseline measures, participants were asked to provide demographic information about their age, gender, ethnicity, country of residence, employment status and education level.

#### Clinical information

Participants reported the name of their skin condition(s) and how long they had had the condition. Participants also indicated whether they were taking any medication for a mental health condition, and if so, reported what medication(s) they were taking and how long they had been taking it.

#### Acceptability measures

As part of assessing the overall acceptability of the intervention, participants’ engagement with the intervention was calculated using Qualtrics data, user logins to the website and data from the overall feedback survey. Treatment completers were defined as participants who logged into the study website during or after week 5 and/or otherwise engaged with the intervention up to at least Session 5. It was hoped that at least 66% of eligible participants would complete the treatment, as this was the median adherence percentage for computerised CBT for depression and anxiety disorders in a meta-analysis [33].

At the end of the study, all participants were asked to provide feedback about which aspects of the intervention were helpful or unhelpful for them and if they could identify any areas for improvement. Open questions with spaces for text responses were used to collect this feedback, as well as an adapted version of the ‘Friends and Family Test’ used in the NHS (see Additional file 1). Participants were also asked to indicate which (if any) of the homework activities they carried out. In line with recommendations that internet interventions monitor negative effects [34], participants were asked whether the intervention exacerbated any existing symptoms or caused any novel symptoms to arise.

To assess the acceptability of the intervention components, each week participants were asked for their feedback on the session they had just completed. Five items from the Website Evaluation Questionnaire [35] and seven items from the Session Impacts Scale [36] were adapted to form this feedback survey, which was the same each week. Website evaluation items used a five-point scale to assess participants’ perceptions about the usability of the study website. Participants were asked to rate their agreement with statements from ‘strongly disagree’ (0) to ‘strongly agree’ (4), for example, ‘I found today’s session easy to use’. Session impact items used a separate five-point scale to assess participants’ perceptions of the personal impact of each self-help session. Participants rated the extent to which statements were true for them, from ‘not at all’ (0) to ‘very much’ (4), for example, ‘As a result of this session, I have realised something new about myself’. In addition, participants had the opportunity to make other comments on the weekly sessions via open-text responses.

#### Feasibility measures

To assess the feasibility of providing the self-help programme online, the percentage of eligible participants who accessed the study website at least once was calculated using Qualtrics data and user logins. To assess the feasibility of providing email guidance, the amount of time that the lead researcher spent facilitating the intervention was recorded. This included registering participants on the study website and responding to individual emails.

#### Outcome measures

The depression subscale of the DASS [29] was used to measure participants’ levels of depression. This consists of 14 items. Participants were asked to rate how much statements applied to them over the past week on a four-point scale from ‘did not apply to me at all’ (0) to ‘applied to me very much, or most of the time’ (3), for example, ‘I couldn’t seem to experience any positive feeling at all’. Scores can range from 0 to 42, with higher scores indicating higher levels of depression. Internal consistency was excellent, with Cronbach’s alpha of 0.96 at baseline.

The Dermatology Life Quality Index (DLQI) [37] was used to measure the impact of participants’ skin conditions. Participants were asked to rate how much their skin condition had affected different areas of their life over the last week on a four-point scale from ‘not at all/not relevant’ (0) to ‘very much’ (3), for example, ‘Over the last week, how much has your skin affected any social or leisure activities?’ There are 10 items and scores can range from 0 to 30. Higher scores indicate a more impaired quality of life. Cronbach’s alpha (0.89) was good at baseline.

The 26-item Self-Compassion Scale (SCS) [38] was used to measure participants’ levels of self-compassion. Participants were asked to rate how often they behave in the manner described in the statements on a five-point scale from ‘almost never’ (1) to ‘almost always’ (5), for example, ‘When I’m going through a very hard time, I give myself the caring and tenderness I need’. The SCS consists of six subscales: self-kindness, self-judgement, common humanity, isolation, mindfulness and over-identification. The mean self-compassion score is obtained by reverse coding items in the self-judgement, isolation and over-identification subscales, calculating a mean for each subscale and then calculating an overall mean, ranging from 1 to 5. Higher scores indicate higher levels of self-compassion. Internal consistency was excellent, with Cronbach’s alpha of 0.90 at baseline.

### Data analysis strategy

The proportions of eligible participants who logged into the study website each week were calculated. Text responses from participants’ weekly and overall feedback were analysed using conventional content analysis [39] to identify helpful aspects of the intervention and areas for improvement. Upon reading the open-text answers for each of the feedback questions, content categories were identified inductively. Categories were modified and added as needed until all comments had been coded. Higher-order themes were identified through a review of the identified categories across the different feedback questions. Descriptive statistics were calculated for the website evaluation and session impact scores of each intervention session. To provide estimates of the intervention’s effect sizes for future research, differences in participants’ psychological outcome measures before and after the intervention were analysed using sign tests. Analyses were carried out with SPSS 26.

## Results

### Participant flow

**Fig. 1:**
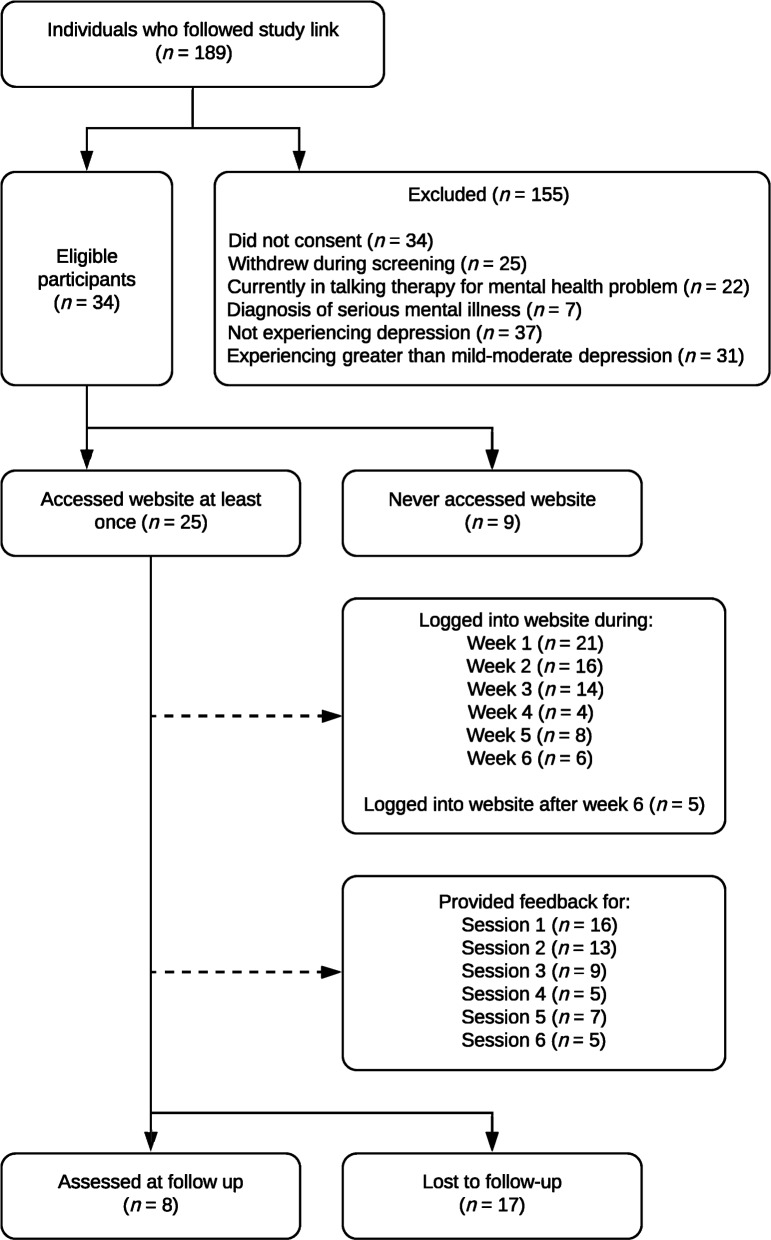
Flowchart of participants throughout the study

Out of 189 people who followed the online link to the baseline survey, only 34 people (18.0%) completed the baseline survey and met the study’s inclusion criteria, and hence were eligible to take part. The remaining 155 people were excluded from the study due to not consenting/completing the baseline survey or not meeting the inclusion criteria. Details are shown in Fig. 1. The study website was accessed at least once by 25 participants. Eight participants gave overall feedback on the intervention and completed the post-treatment outcome measures.

### Baseline characteristics

The majority of participants who were registered on the study website (*n* = 34) were female (91.2%), located in the UK (91.2%), in employment (64.7%), and reported their ethnicity as ‘white’ (82.4%). The mean age was 38.2 years (SD = 11.5, range 21–61) and the mean duration of skin condition was 23.9 years (SD = 15.1, range 1–51). All participants who reported taking psychotropic medication (*n* = 12) were taking an anti-depressant, and the average duration of this medication was 5.74 years (SD = 5.84). The majority of participants (85.3%) had eczema or psoriasis, which are common pruritic skin conditions. Other demographic data and baseline scores on outcome measures are presented in Table 2. Within the baseline outcome measure data from the 34 eligible participants, missing data were minimal, at 0.002%. Participants’ scale means were used to replace missing items.

**Table 2:** Baseline demographic and clinical characteristics of participants

Characteristic	Treatment completers (*n* = 13)	Non-completers (*n* = 21)	Total sample (*n* = 34)
Age, years (mean, SD)	43.77 (13.22)	35.24 (9.67)	38.50 (11.75)
Gender (*n*)			
Female	11	20	31
Male	2	1	3
Ethnicity (*n*)			
White or White British	13	15	28
Mixed heritage	0	2	2
British African	0	1	1
British Pakistani	0	1	1
Japanese	0	1	1
Not specified	0	1	1
Employment status (*n*)			
Employed/self-employed	10	12	22
Full-time homemaker/carer	0	4	4
Unable to work	2	1	3
Student	1	1	2
Retired	0	1	1
Unemployed	0	1	1
Not specified	0	1	1
Highest qualification level (*n*)			
GCSE or equivalent	2	3	5
A-level or equivalent	0	4	4
Level 4 or 5 qualification	2	0	2
Bachelors’ degree	4	10	14
Masters’ degree	4	3	7
Postgraduate degree	1	1	2
Skin condition(s)^a^ (*n*)			
Eczema	5	11	16
Psoriasis	8	5	13
Autoimmune conditions	3	1	4
Actinic cheilitis	1	0	1
Contact allergies	1	0	1
Chronic urticaria	0	1	1
Lichen planus	0	1	1
Nodular prurigo	1	0	1
Pemphigus vulgaris	0	1	1
Vitiligo	0	1	1
Psychotropic medication use (*n*)			
No	8	14	22
Yes	5	7	12
DASS-D score (mean, SD)	15.15 (2.97)	14.38 (2.84)	14.68 (2.87)
DLQI score (mean, SD)	16.92 (7.91)	11.90 (6.16)	13.82 (7.21)
SCS score (mean, SD)	2.46 (0.59)	2.63 (0.39)	2.56 (0.48)

*Note. DASS–D *Depression subscale of Depression Anxiety Stress Scales, *DLQI* Dermatology Life Quality Index, *SCS* Self-Compassion Scale

^a^Six participants had more than one skin condition

### Acceptability outcomes

#### Participant engagement

Out of the 34 participants registered on the study website, 11 participants were still engaged with the self-help programme (i.e. logged into the study website) during or after their fifth week of the intervention. Six of these participants provided overall feedback via the post-treatment survey. Overall feedback was also provided by two further participants, whose answers indicated that they had completed the exercises up to at least Week 5 or printed all the webpages at an earlier date. These two participants were therefore also classed as treatment completers. Accordingly, the total number of treatment completers was 13, with post-treatment data provided by eight of these. Retention of eligible participants (*n* = 34) was therefore 38.2%, while retention of participants who began the programme (*n* = 25) was 52%. In both cases, participant retention was less than the hoped-for 66%. Characteristics of participants who completed the intervention and those who did not are presented in Table 2.

#### Overall feedback

Of the eight participants who provided overall feedback, three reported that they would be ‘extremely likely’ to recommend the Compassion for Skin Conditions self-help programme to family or friends if they needed similar help. Four more participants reported that they would be ‘likely’ to do so, and one reported that it was ‘neither likely nor unlikely’. Therefore, 87.5% of participants who provided feedback would recommend this self-help programme to others in its current format. Content analysis of open-text responses indicated that treatment completers perceived the intervention as easy to use and that they appreciated the skin-specific nature of the programme. Participants were divided on whether they found the soothing rhythm breathing helpful and found noticing negative thoughts less helpful, or vice versa. The compassionate imagery was also noted as being challenging, but all participants perceived there to have been a benefit in taking part in the programme and most expressed gratitude at having been able to do so. Three participants noted a negative effect of taking part in the programme: one felt more self-conscious due to reading examples of worries that she had not had before, and two noted emotional discomfort due to an increased awareness of negative thoughts, but one of these participants further noted that this temporary discomfort had been beneficial overall. Further details of the content analysis are provided in Additional file 2.

Two suggestions for improvement of the intervention related to the intensity of the programme: there was one suggestion for daily email prompts about homework activities and one suggestion for longer between sessions and a follow-up session at a later date. Other suggestions were idiosyncratic: there was one suggestion for even more skin-specific examples and advice, particularly around the physical aspects of living with a skin condition, one suggestion to provide samples of skin products, and one minor typographic suggestion.

**Fig. 2:**
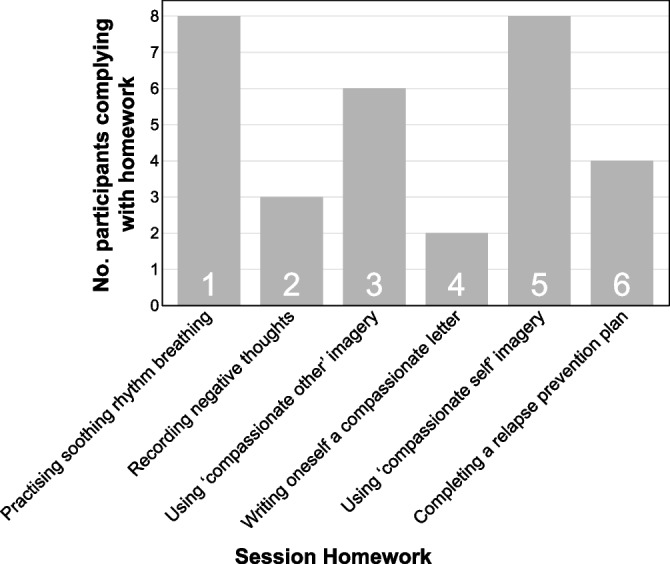
Self-reported homework compliance by session

Figure 2 shows participants’ self-reported compliance with the recommended homework from each session. The breathing and imagery exercises (from Sessions 1, 3 and 5) were practised by more participants than the written homework exercises (from Sessions 2, 4 and 6).

#### Weekly feedback measures

**Fig. 3:**
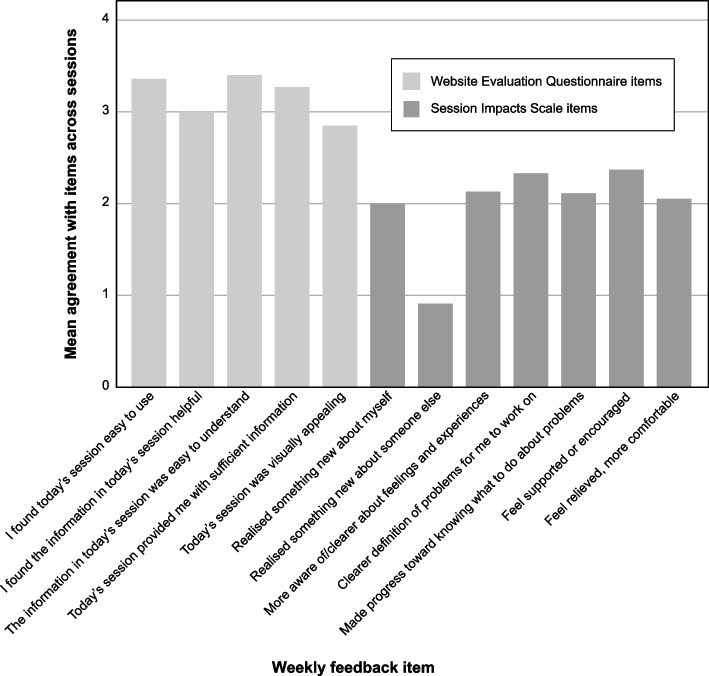
Mean agreement with weekly feedback items for the Compassion for Skin Conditions intervention *Note.* For Website Evaluation Questionnaire items, 0 = “strongly disagree”, 1 = “disagree”, 2 = “neutral”, 3 = “agree”, 4 = “strongly agree”. For Session Impacts Scale items, 0 = “not at all”, 1 = “slightly”, 2 = “somewhat”, 3 = “pretty much”, 4 = “very much”

Means for the website evaluation and session impact items were computed for the self-help intervention as a whole. These are shown in Fig. 3. Overall, participants evaluated the website positively: on average, agreeing with statements that it was easy to use and helpful, but less positive about the visual appeal of the website. Participants mostly agreed that the intervention had had “somewhat” of an impact across the different items, with the exception of the item “I have realised something new about someone else”, which was rated between “not at all” and “slightly”.

**Fig. 4:**
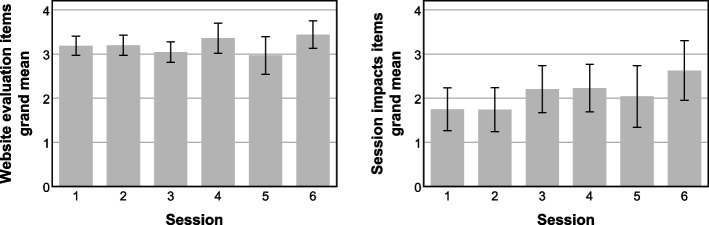
Grand means of weekly feedback items by session *Note.* Error bars represent one standard deviation

Feedback scores were also examined by session to investigate differences between sessions. Website evaluation and session impact grand means were calculated for each session. Results are shown in Fig. 4. Overall, website evaluations were fairly consistent across sessions, with grand means ranging from 2.97 to 3.44 on the 0–4 scale. Ratings of session impacts were less consistent, ranging from 1.74 to 2.63, and increasing across sessions.

Open-text responses within the weekly feedback survey indicated that participants were aware of finding some of the exercises challenging and needing to practise to benefit from them. The only negative comments about the programme reflected those that were captured in the overall feedback: one participant struggled with the breathing practice, one did not find the session on negative thoughts helpful, and one felt that more focus on the physical aspects of skin conditions would be helpful. Participants reported feeling more positive and being more aware of their difficulties and helpful strategies after the sessions. For some participants, the session content reminded them of previously learnt coping strategies, such as breath awareness or visualisations. Participants also commented that they liked the skin-specific nature of the programme and found the audio resources and links to further resources helpful.

### Feasibility outcomes

Logins to the study website showed that 25 of the 34 eligible participants (73.5%) successfully accessed the intervention. However, of the nine participants who did not successfully log into the study website, only two attempted to do so: seven participants never tried to log in.

Facilitating the intervention required only around six and a half hours of the researcher’s time: registering participants on the study website took a total of 68 min, while supporting participants through the intervention took 5 h and 17 min. Dividing the total time spent facilitating the intervention by the number of participants gave an average of 11.3 min of researcher time spent per eligible participant who began the study, and 48.1 min per participant who provided post-treatment data.

### Depression, quality of life and self-compassion outcomes

Outcome measure data from treatment completers was analysed with non-parametric tests, as the sample size was small (*n* = 8). Tests of normality indicated that the distribution of the pre-post differences on the SCS was positively skewed (*z* = 2.41) and leptokurtic (*z* = 2.71). The sign test was therefore the most appropriate test to use for these data, as the positive skew meant that the distribution was not symmetric and therefore did not meet the assumptions for the Wilcoxon signed-rank test. The distributions of changes on the DASS-D and DLQI were approximately normal, but the sign test was also used for these data for consistency. Results are shown in Table 3. Median depression, quality of life, and self-compassion scores improved between pre- and post-intervention, although only the change in the quality of life score reached statistical significance at *p* < 0.05. Effect sizes were *g* = 0.25 for depression, 0.38 for quality of life, and 0.25 for self-compassion, all of which are large effect sizes according to Cohen [40].

**Table 3:** Sign test results for psychological outcome measures (*n* = 8)

Measure	Pre-treatment median	Post-treatment median	Increases (*n*)^a^	Decreases (*n*)^b^	Ties (*n*)^c^	*P*^*d*^	Effect size (Cohen’s *g*)
DASS-D	14.50	8.50	2	6	0	.15	0.25
DLQI	14.00	8.00	1	7	0	.04	0.38
SCS	2.68	3.16	6	2	0	.15	0.25

*Note. DASS–D* Depression subscale of Depression Anxiety Stress Scales, *DLQI* Dermatology Life Quality Index, *SCS* Self-Compassion Scale

^a^Pre-treatment score < post-treatment score. ^b^Pre-treatment score > post-treatment score. ^c^Pre-treatment score = post-treatment score. ^d^Exact significance (1-tailed)

## Discussion

This study aimed to investigate the acceptability and feasibility of a novel compassion-based self-help intervention for depression in people with skin conditions. Feedback gathered suggested that the content and format of the self-help programme were acceptable to users in its current format, although improvements could be made. This raises the question as to why the attrition rate was so high, at 61.8%, between eligible participants being registered on the study website and completing the intervention. Some attrition is to be expected for any intervention as changes in life circumstances, such as ill health or competing demands on people’s time, can prevent people from continuing with non-essential plans. However, in the absence of data about why people dropped out, it is impossible to know the proportion of participants for whom this was the case. It is worth noting that high attrition rates are common for internet-based interventions [33].

A number of treatment factors are known to affect engagement, adherence and attrition for online CBT interventions. Support from a practitioner improves adherence, particularly when the practitioner initiates contact rather than simply being available in case of problems [41]. In the current study, quasi-personal contact was provided by personalised, but automated, guidance emails. These contained the participant’s name and related to the specific intervention session that they were due to complete that week, but were not linked to logins to the study website. However, no participants took up the offer of contacting the researcher because of difficulties with the intervention, although some participants contacted the researcher for administrative reasons (e.g. login difficulties). Therefore, most participants did not make use of the support available. Future studies could include telephone contact with participants prior to beginning the intervention, which would increase the amount of support participants receive and also provide an opportunity to address any concerns, misunderstandings or barriers to treatment [41]. In addition, future studies could include information about what to expect from the intervention as part of a ‘cooling off’ period for eligible participants, that is, between screening and the beginning of the intervention. This could consist of a ‘taster’ session, information that addresses common concerns or misunderstandings about online self-help, or quotes from previous participants to enhance treatment credibility [41]. To improve understanding of attrition in online interventions, future studies could also contact people who have dropped out with a single ‘inline’ email question (where the survey question is in the body of the email itself) about the reason for their discontinuation. When it is easy to provide feedback, participants are more likely to do so [42], so it may be helpful to provide common reasons for discontinuation as multiple-choice options.

Website evaluation scores and open text comments showed that participants found the website easy to use and that the content was helpful and easy to understand. Future studies could further improve the intervention by offering participants more flexibility about how they use the programme. This should include choices about how often email reminders are sent and at what pace participants go through the programme. Asking participants to specify these preferences, and enabling options accordingly, would help tailor the intervention to individuals’ needs. It may also be beneficial to allow some choice about the order of sessions: as there was a divide between participants about whether they found the written vs. breathing/imagery exercises most helpful, allowing participants some flexibility about when (or even if, based on previous experiences) they do these exercises might help keep participants engaged with the intervention. The programme could also be amended to include information for people who are encountering blocks and resistance to the compassion-based exercises [31]. However, such information need not be presented as part of the main body of the intervention, but as an optional extra, thus allowing participants to tailor the intervention to their needs. This would also be consistent with recommendations that internet interventions allow people to access more in-depth information as desired [43].

Results also showed that it was feasible to provide a guided online compassion-based intervention, requiring relatively little researcher time during the data collection period. This suggests that the intervention would be feasible to facilitate as part of a larger-scale trial in the future without requiring extensive resources. The effects of the intervention on outcome measures were promising, with treatment completers showing improvements in depression, self-compassion and dermatology quality of life. Although hypothesis testing was not an aim of the current study, results suggest that compassion-based self-help holds promise as a treatment for depression in people with skin conditions, which is in line with previous research [23].

### Strengths and limitations

An important strength of this study was that only participants who reported mild–moderate symptoms of depression at baseline were included. This meant that the acceptability and feasibility of the intervention were explored within the target population, which added external validity to the findings. Furthermore, treatment completers were participants with a variety of skin conditions, meaning that the intervention successfully catered to the needs of people with heterogeneous skin conditions. In contrast with other self-help resources that are tailored for people with specific skin conditions such as psoriasis [5, 6], the current intervention is also a suitable resource for people with rarer skin conditions who may otherwise find relevant skin-specific self-help unavailable. However, there were also a number of limitations to the study that should be noted. First, although all eligible participants were invited to provide overall feedback, regardless of how much of the programme they undertook, the only participants who chose to do so were those who remained engaged with the intervention until at least week 5. This means that the overall feedback was likely to be positively biased, as it was only completed by participants who, presumably, perceived the programme to be beneficial. Weekly feedback measures, which avoid retrospective bias, were also used to gain a more accurate picture of how the intervention was perceived across participants. However, even these weekly feedback scores may have been affected by dropouts: the slight increase in impact scores across sessions probably reflects the attrition of participants who were perceiving less benefit from the intervention. Nevertheless, the weekly feedback for the earlier sessions indicates that the website was positively evaluated by and had at least some impact on all participants who began the intervention.

Second, the large majority of eligible participants (91.2%) were female. This suggests that the intervention appealed most to women, which limits generalisability. It is unclear whether the gender bias of the sample was specific to this intervention, or whether it was part of wider issues about the role of gender in help-seeking behaviour and treatment preferences: compared to women, men are less likely to seek help for depression (e.g. [44]) and tend to be less interested in the psychosocial aspects of online depression resources [45]. Further research is required to investigate how to engage men in accessing self-help resources, and whether the intervention could be made more appealing to men.

Finally, while appropriate for a feasibility study, the uncontrolled pre–post design of the study limits the conclusions that can be made about the effects of the intervention. Outcome measures were used in the current study to provide estimates of effect sizes to inform future studies, and results indicated that participants who completed the intervention had improved depression, self-compassion and quality of life compared to baseline. However, without a control group and randomisation, strong causal inferences cannot be drawn.

## Conclusions

This study demonstrated that an online, six-week, guided self-help intervention based on compassion can be provided to people with skin conditions and symptoms of depression. The intervention holds potential as a low-intensity treatment for depression in people with skin conditions, as user feedback indicated the content and format of the intervention were perceived as acceptable and ideas for further improvement have been identified. In addition, improvements in depression, self-compassion and skin-related quality of life were observed for treatment completers. Further research on the use of compassion-based self-help for people with skin conditions is warranted. This could usefully consist of further intervention development work to increase participants’ engagement with compassion-based self-help, and a randomised controlled trial to further investigate its effects in people living with skin conditions.

### Supplementary Information

**Additional file 1.** The weekly feedback questions and the overall feedback questions used in this study.

**Additional file 2.** Details of the content analysis of qualitative feedback given by treatment completers.

## Data Availability

The datasets generated during the current study are not publicly available as participants did not provide written consent for their data to be shared publicly. Data that support the findings of this study are available from the corresponding author upon reasonable request.
