# Supplementary material for: Online compassion-based self-help for depression in people with skin conditions: a feasibility study
Source: Pilot Feasibility Stud. 2024 Apr 16;10:63. doi: 10.1186/s40814-024-01486-4 (PMC11020170; doi:10.1186/s40814-024-01486-4)
Supplement: Supplementary file 1 — Additional file 1. The weekly feedback questions and the overall feedback questions used in this study. [file 40814_2024_1486_MOESM1_ESM.docx]

# Additional file 1

## Weekly Feedback Questions

Please answer the following questions about the self-help session you have just completed (<title of webpage>).

|  | Strongly disagree | Disagree | Neutral | Agree | Strongly agree |
| --- | --- | --- | --- | --- | --- |
| I found today’s session easy to use. | ⭘ | ⭘ | ⭘ | ⭘ | ⭘ |
| I found the information in today’s session helpful. | ⭘ | ⭘ | ⭘ | ⭘ | ⭘ |
| The information in today’s session was easy to understand. | ⭘ | ⭘ | ⭘ | ⭘ | ⭘ |
| Today’s session provided me with sufficient information. | ⭘ | ⭘ | ⭘ | ⭘ | ⭘ |
| Today’s session was visually appealing. | ⭘ | ⭘ | ⭘ | ⭘ | ⭘ |

As a result of this session:

|  | Not at all | Slightly | Somewhat | Pretty much | Very much |
| --- | --- | --- | --- | --- | --- |
| I have realised something new about myself. | ⭘ | ⭘ | ⭘ | ⭘ | ⭘ |
| I have realised something new about someone else. | ⭘ | ⭘ | ⭘ | ⭘ | ⭘ |
| I am more aware of / clearer about feelings and experiences. | ⭘ | ⭘ | ⭘ | ⭘ | ⭘ |
| I have a clearer definition of problems for me to work on. | ⭘ | ⭘ | ⭘ | ⭘ | ⭘ |
| I have made progress toward knowing what to do about problems. | ⭘ | ⭘ | ⭘ | ⭘ | ⭘ |
| I feel supported or encouraged. | ⭘ | ⭘ | ⭘ | ⭘ | ⭘ |
| I feel relieved, more comfortable. | ⭘ | ⭘ | ⭘ | ⭘ | ⭘ |

Are there any specific comments you would like to make about today’s session?

|  |
| --- |

## Overall Feedback Questions

We would like you to think of your recent experience with using the Compassion for Skin Conditions self-help programme (website, downloadable resources, and email support over six weeks).

How likely are you to recommend the Compassion for Skin Conditions self-help programme to friends and family if they needed similar help?

| Extremely likely | Likely | Neither likely nor unlikely | Unlikely | Extremely unlikely | Don’t know |
| --- | --- | --- | --- | --- | --- |
| ⭘ | ⭘ | ⭘ | ⭘ | ⭘ | ⭘ |

What was good about the Compassion for Skin Conditions self-help programme?

|  |
| --- |

What would have made the Compassion for Skin Conditions self-help programme better?

|  |
| --- |

Has taking part in the Compassion for Skin Conditions self-help programme been beneficial for you? If yes, in what way(s)?

|  |
| --- |

Did you experience any negative effects while taking part in the Compassion for Skin Conditions self-help programme (e.g., worsening of existing difficulties or new difficulties arising)? If yes, what negative effect(s) occurred?

|  |
| --- |

During the self-help programme, which of the following activities did you do in addition to the website sessions (i.e., homework activities)? Tick all that apply.

| Practising soothing rhythm breathing | ⭘ |
| --- | --- |
| Recording negative thoughts | ⭘ |
| Using ‘compassionate other’ imagery | ⭘ |
| Writing a compassionate letter to yourself | ⭘ |
| Using ‘compassionate self’ imagery | ⭘ |
| Completing a relapse prevention plan | ⭘ |

Which part of the Compassion for Skin Conditions programme was the most helpful/valuable for you?

|  |
| --- |

Which part of the Compassion for Skin Conditions programme was the least helpful/valuable for you?

|  |
| --- |

Is there anything else you would like us to know about your experience of using the Compassion for Skin Conditions self-help programme?

|  |
| --- |
