# Supplementary material for: Online compassion-based self-help for depression in people with skin conditions: a feasibility study
Source: Pilot Feasibility Stud. 2024 Apr 16;10:63. doi: 10.1186/s40814-024-01486-4 (PMC11020170; doi:10.1186/s40814-024-01486-4)
Supplement: Supplementary file 2 — Additional file 2. Details of the content analysis of qualitative feedback given by treatment completers. [file 40814_2024_1486_MOESM2_ESM.docx]

**Additional file 2**

Table A1. Content analysis of qualitative feedback given by treatment completers (n = 8)

| Higher order theme | Content category | Frequency | Example comment | |
| --- | --- | --- | --- | --- |
| ***Good aspects of the programme*** | | | | |
| The programme is easy to use | Easy to use | 5 | The programme was easy to use, well-written, concise and the website was easy to navigate. The aims of the programme seemed very clear. | |
| The skin specific nature of the programme is helpful | Normalising | 2 | The whole thing was helpful and to know your (sic) not alone with it. | |
|  | Mind-skin link | 1 | It reminded me how much of a role stress plays in my eczema and that looking after my mental health is a core part of looking after my skin | |
|  | Raises awareness and changes thinking patterns about skin | 1 | Makes you think about the way you think about your skin condition. Highlights the problems you have with your skin which you may not have realised before. Changes your way of thinking about your skin condition. | |
| Including a variety of exercises is useful | Variety of exercises | 1 | Really easy to follow with lots of great exercises to do! | |
| ***Areas for improvement*** | | | | |
| The programme is acceptable in its current format | Nothing needs to change | 3 | Nothing really, it was very easy to access. | |
| More flexibility / choices about how to use the programme would be beneficial | Daily emails | 1 | I printed off all the sheets but maybe would have found daily email prompts of specific things to do helpful | |
|  | Longer between sessions/follow-up | 1 | 2 weeks apart between sessions, difficult to keep up when other things are going on in your life. 6 weeks doesn’t seem long enough to make a lasting difference. May be good to revisit and have a refresher session a few weeks after the end | |
| More focus on impact of skin condition, especially physical aspects | More focus on impact of physical symptoms | 1 | More advice on how to cope with physical aspects damaging any positive thought | |
| Need to manage expectations | Skincare samples | 1 | Some free samples of creams or ointments | |
| Visual appeal of the website | Typography changes | 1 | Slightly less text or split over the page slightly more so it wasn’t as grouped together. | |
| ***Benefits of taking part*** | | | | |
| Taking part in the programme was beneficial | Raised awareness of attitude towards oneself | 4 | Yes, made me think of my thinking in a different way. | |
|  | Acceptance of skin condition | 2 | Yes it has made me more accepting of my skin condition. | |
|  | Increased self-care and improved skin | 1 | Yes, I am focusing much more on looking after my overall wellbeing and I think that’s reducing my skin flares. | |
|  | Raised awareness of mind-skin link | 1 | I understand my skin and how I am feeling can make it worse. | |
|  | Reduced isolation and increased hope | 1 | Yes realising I’m not alone and things can change | |
| ***Negative effects of taking part*** | | | |  |
| Taking part in the programme was beneficial | No negative effects | 5 | No. | |
| Taking part in the programme can cause some distress | Emotional discomfort | 2 | No not really. It can be uncomfortable when negative feelings arise but I feel overall these have been beneficial. I’m more aware of how I treat myself and how I’ve had the tendency to focus on negative issues to do with my skin, getting stressed and quite likely, making things worse. | |
|  | Increased self-consciousness | 1 | Sometimes I hadn’t thought about the worries before like what other people are thinking while I’m out in general/at work/etc and made me feel a bit conscious that people are looking at me. | |
| ***Most helpful aspect of the programme*** | | | |  |
| Including a variety of exercises is useful | Soothing rhythm breathing | 4 | I really loved the rhythm breathing, I use it all the time now. | |
|  | Noticing negative thoughts | 2 | Session 2, Thoughts & Feelings. As I’ve said, this session really made me think & it was quite a shock to appreciate how harshly I’ve been treating myself. | |
| The programme is acceptable in its current format | All/unspecified | 2 | Learning about how to be kind to yourself. | |
| ***Least helpful aspect of the programme*** | | | |  |
| The programme is acceptable in its current format | Nothing identified | 2 | All of the programme was helpful! | |
| Including a variety of exercises is useful | Compassionate imagery | 2 | The imagery but that’s just because I found it difficult to do. | |
|  | Recording negative thoughts | 2 | Recording negative thoughts - I know in the past that has made me dwell on them if I write them down. | |
|  | Audio tracks | 1 | Listening to the recordings. | |
|  | Soothing rhythm breathing | 1 | Breathing. Can never get the hang of it. | |
| ***Other comments*** | | | |  |
| Taking part in the programme was beneficial | Gratitude | 6 | It was great to be part of something simple but so effective, thank you. | |
